# Severe psychogenic tremor of both wrists in a 13-year-old girl treated successfully with a customized wrist brace: a case report

**DOI:** 10.1186/1752-1947-5-158

**Published:** 2011-04-20

**Authors:** Elisabeth Sauerhoefer, Caroline Schafflhuber, Oliver Kratz

**Affiliations:** 1 Department of Child and Adolescent Mental Health, University of Erlangen, 91054 Erlangen, Germany

## Abstract

**Introduction:**

Psychogenic movement disorders in childhood have been little researched. As there are few courses of treatment which have been evaluated, further examination and case studies about the treatment and clinical course of this rare occurrence of severe psychogenic tremor in childhood and adolescence are much needed.

**Case presentation:**

A 13-year-old Caucasian girl with tremor in both wrists, severe enough to prevent her from attending school, was sent to our hospital. After a complete neurological and psychiatric examination, in-patient child-psychotherapeutic treatment was started, with careful consideration given to both chronic and acute stress factors which constitute her performance and exam anxiety in school as well as the girl's parents' conflicted relationship. With the aid of a customized wrist brace our patient was able to go to school and write despite the presence of a marked tremor, which in turn reduced her avoidance behavior and exam anxiety. By the end of her in-patient treatment, the tremor was still noticeable, but markedly reduced in severity (reduction 80%). Two weeks after she was discharged from hospital, the tremor had completely disappeared.

**Conclusion:**

After careful clinical diagnostics, this kind of dissociative disorder should be treated appropriately with age-adapted cognitive-behavioral therapy to achieve positive and lasting benefits.

## Introduction

Dissociative disorder is characterized by the partial or complete lack of the normally integrated functions of memories about the past, identity, perception of the environment, and control of physical movements. Diagnostic criteria include lack of a medical condition which would explain the typical characteristics of this syndrome and a conclusive temporal relation between the onset of dissociative symptoms and stressful events, problems or needs of the patient.

The International Classification of Diseases (ICD-10, chapter F: mental and behavioral disorders) lists in section F 44: dissociative disorders with impaired consciousness (amnesia, fugue, stupor, trance); dissociative motor disorders (paralysis, astasia, abasia, ataxia, dysarthria, aphonia, shaking, tremor); dissociative convulsion; and dissociative anesthesia/sensory loss.

Due to a lack of systematic studies, no accurate prediction regarding the rate of dissociative disorder during childhood and adolescence can be made. According to population-based prevalence studies using structured clinical interviews, lifetime prevalence of dissociative disorder is estimated to be between 2-7% [1,2] in North America.

## Case presentation

One week prior to her first visit to our clinic, the 13-year-old Caucasian girl noticed muscle twitching in her right (dominate) hand or a fluttering sensation. The muscle twitching resolved itself spontaneously after one hour. On the following day immediately before taking an exam at school, she experienced a severe tremor in her right hand which spread to her left hand and persisted. Immediately after leaving school, the girl was hospitalized at the neurology ward of a pediatric clinic where she underwent a complete medical check-up. After a thorough examination, no neurological or medical explanation for the symptoms was found. A physiotherapist asked her to "stop pretending", a notion that caused prolonged anger and sadness in her.

Our patient agreed to be transferred to the child and adolescent psychiatric department. The tremor continued after admission and it could only be interrupted by either grasping an object in her hand or clenching her hand. She was not able at this time to either write or perform activities requiring fine motor skills (for example, cutting something with a knife). In order to fall asleep our patient had to either lie on her hands or hold an object in her hands.

### Personal and family history

Our patient was attending the 7^th ^grade in an academic secondary school and was in danger of failing academically. There was no history of psychiatric, neurologic or other severe somatic disorder in her past. She lived with her parents, who had been fighting regularly for years. The question of a separation was an issue between them, but they reported never having talked about it openly with their daughter.

### Investigations

Our patient was appropriately developed for her age. Her cardio-pulmonary status and the results of examinations of her head, neck, abdomen, skin and genitals were all normal.

The finger to nose test, motor proficiency tests, pupillary reactions and eye movement tests were all normal. Her reflex status showed normal tonicity for both sides of her body, no meningism was present, gross strength was normal and sensitivity was normal for both sides of her body. During the examination the tremor ceased. At rest a low frequency hand tremor could be observed (approximately 5/s) in both hands. By the end of the physical examination the tremor changed from a low frequency tremor to a diadochokinetic tremor (Additional file 1).

Blood tests revealed no abnormalities, including thyroid stimulating hormone. Ceruloplasmin, serum copper and urine copper levels were taken and an ophthalmological evaluation was performed to rule out Wilson's disease. The results of cranial magnetic resonance imaging and an electroencephalogram were normal, as were results of a cerebrospinal fluid abdominal ultrasound.

Psychiatric diagnostic testing included German versions of the Anxiety Questionnaire for Children, which showed a high tendency toward social desirability and performance anxiety (schoolwork). Our patient showed above average intelligence (HAWIK-IV, German version of WISC: IQ = 124). A scale to measure depression in children showed no marked symptoms of depression. For observation purposes and for a more precise diagnosis, the patient was filmed.

Our patient's symptoms were noticeably lessened when her attention was on something else, for example on mental arithmetic. Concentrated on her tremor resulted in it becoming intensified. During her examination, the tremor fluctuated greatly. An entrainment test [3] showed that when our patient tapped a rhythm with her contra-lateral hand, the tremor matched this new rhythm and at times ceased altogether.

### Treatment

Our patient completed a comprehensive medical-behavioral therapy which included individual and group therapy sessions (client-centered in orientation) and anxiety-reducing techniques, especially in the area of performance anxiety. She also learned progressive muscle relaxation techniques (PMR).

One important aspect of the treatment was to work with our patient to help her understand what triggered her tremors and kept them from diminishing. In particular, her performance anxiety and her avoidance of challenges relating to school can be seen as factors involved in the disorder. Every day, our patient trained to write for about a quarter of an hour. At the beginning of her stay in our clinic, her motivation to go back to school or to write on her own was very low (Additional file 2).

**Figure 1:**
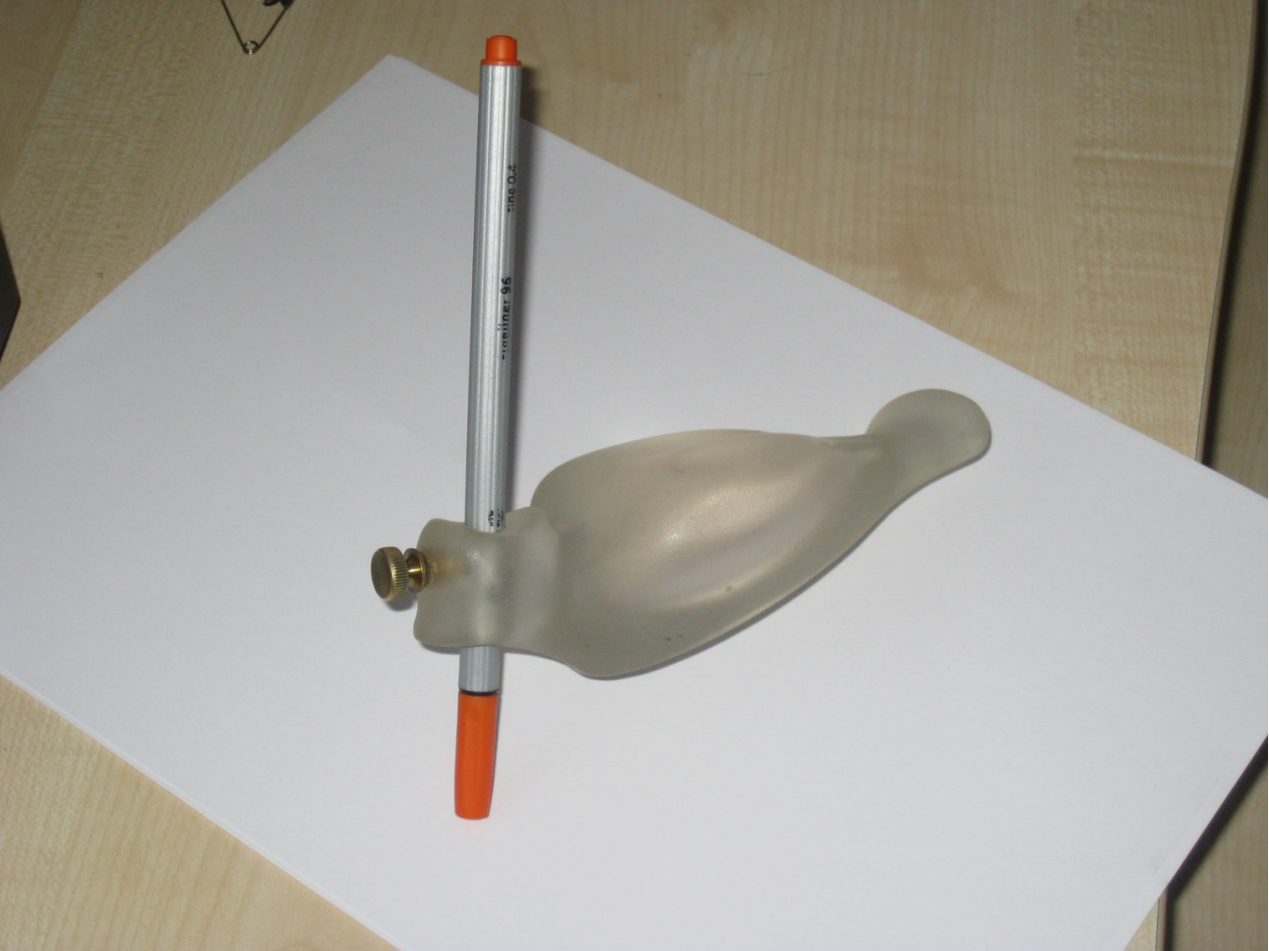
**Wrist brace**.

A customized wrist brace was adapted to her hand, making it easier for her to write (Figure 1).

From this time she was sent to school regularly and had to participate in every exam. Our patient participated in private lessons because of great difficulties in certain school subjects, which helped her to overcome her anxiety concerning school. The tremor intensity was strongly related to her individual stress level and parental conflicts. By the end of the period of hospitalization, our patient's tremor was still present, but noticeably reduced in degree (reduction of 80%). Despite the presence of a slight tremor, the girl was able to write with the aid of the customized wrist brace (Additional file 3).

After completion of the therapy, our patient described what had helped her most in dealing with the symptoms of her tremor. First was to become more physically active rather than retiring alone to her room to listen to music. In doing so, she was able to cope with her symptoms both on a physical and on a social (interpersonal) level as she had been taught in therapy. Secondly, identifying and relating her feelings to others. Thirdly, learning and practicing a relaxation method to reduce her stress, in this case PMR, which had initially proven difficult for her, but which she was able to master in the end. Finally, attending regular physical therapy sessions helped in ameliorating her symptoms.

### Outcome and follow-up

Our patient had her first check-up two weeks after being discharged from our clinic, after a total duration of in-patient hospitalization of three months. At this time the tremor had ceased altogether and reappeared only about once every two months in extremely stressful situations. She reported that her parents were still living together, but planned to split up. The total duration of her treatment was nine months (Additional file 4).

## Discussion

No definite recommendations for treatment of pronounced psychogenic tremor in adolescents were found in the literature on this topic. Due to the complex nature of this disorder, the difficulty of diagnosis and the often prevailing convictions of the patient that the cause of their suffering is physical in nature, this disorder often goes unrecognized and is not properly treated.

To lend further credence to the diagnosis of psychogenic tremor, typical signs of the disorder could be observed in our patient. For the most part the symptoms of dissociative (conversion) disorder begin abruptly and without being related to a specific event, as happened in our case.

When treating these disorders, it is of utmost importance to intervene quickly as this has considerable implications for the prognosis. The prognosis was found to be dependent on the amount of time which elapses between the time symptoms become evident and adequate treatment begins. The shorter this time period was, the better the prognosis. Another factor was the success of the treatment during the in-patient treatment. In patients whose symptoms decreased during the initial phase of the hospital stay, 96% of them saw definite positive outcomes, whereas in patients that had no initial decrease of symptoms, only 30% later had positive outcomes [4].

In our case, our patient was admitted only several days after the onset of symptoms. After a thorough physical exam revealed no neurological etiology, our patient was admitted to our department for treatment with a case of suspected psychogenic tremor.

Treatment of dissociative disorder should include the non-judgmental acceptance of the presenting symptoms, or rather the avoidance of accusing the patient of simulation, and respect for the somatic problems of the patient.

In our case, after the physiotherapist suggested the girl was pretending to have the tremor, the tremor was aggravated. Afterwards the girl told us that she was really disappointed and angry because the physiotherapist did not accept her symptoms.

The use of symptom-oriented approaches, both directly and indirectly, seem to be of particular benefit in the treatment of dissociative disorder. Direct therapy would include physical therapy exercises, for example, gradually increasing strain on the body part in question through the use of crutches, physiotherapy and manual therapies with the goal of symptom reduction under the self-control of the patient, which enables them to save face and "escape with honor" [5].

The success of cognitive-behavioral therapy requires the motivation and the co-operation of the patient. Positive feedback through the use of video recordings or clear signs of improvement, which increase the patient's motivation, can be integrated into cognitive behavioral therapy [4]. Video recordings were also used extensively in the girl´s case, to show her the reduction of her symptoms during the course of her hospitalization.

The goal of cognitive-behavioral approaches is to obtain an understanding of the psychological causes of the disorder, in this case conflicted relations in the family, feeling overwhelmed at work or at school due to exam anxiety, to reduce avoidance behavior, which is seen as unwanted behavior, and to positively reinforce desired behavior.

With the aid of a customized wrist brace, the girl was able to return to school and write despite the presence of a marked tremor, which in turn reduced her avoidance behavior and hence noticeably reduce her exam anxiety.

## Conclusion

After comprehensive clinical diagnostics, our patient's psychogenic tremor was treated with cognitive-behavioral therapy, which offers a pragmatic approach to reduce symptoms. This treatment should be adapted to the needs of the patient and typical mistakes like accusing the patient of simulation should be avoided. With the aid of a customized wrist brace, the girl was able to attend school and to write despite the presence of a marked tremor. This reduced her avoidance behavior and hence also her exam anxiety.

Further examination and case studies about the treatment and clinical course of this rare occurrence of severe psychogenic tremor in childhood and adolescence are much needed.

## Consent

Written informed consent was obtained from the patient´s parents for publication of this case report, accompanying images and videos. A copy of the written consent is available for review by the Editor-in-Chief of this journal.

## Competing interests

The authors declare that they have no competing interests.

## Authors' contributions

ES, CS and OK were involved in the diagnosis and therapy of the patient.

ES drafted the manuscript. OK and CS revised the manuscript. All authors read and approved the final manuscript.

## Supplementary Material

Additional file 1 **Tremor before therapy started**. Video Click here for file

Additional file 2 **Writing before therapy started**. 2: Video Click here for file

Additional file 3 **Writing with wrist brace**. Video Click here for file

Additional file 4 **Status after therapy**. Video Click here for file
